# Invisible wounds of the Israel–Gaza war in Australia

**DOI:** 10.5694/mja2.52168

**Published:** 2023-11-14

**Authors:** Susan J Rees, Batool Moussa

**Affiliations:** ^1^ Discipline of Psychiatry and Mental Health University of New South Wales Sydney NSW

**Keywords:** Post‐traumatic stress disorder, Refugees, Stress

Among the many dire consequences of the Israel–Gaza war that began in October 2023, the impact on the mental health of populations living in multicultural Western countries is significant and should not be overlooked.^1^ The psychosocial reverberations of the conflict are felt in societies throughout the world, embodying unique characteristics of trauma and adding to the complexity of the mental health risk for people living in Western countries. The threat to mental health status is higher for those who have had family members killed, harmed or gone missing, and for those with previous exposure to war, including in Lebanon, Iraq and Syria. The level of stress has been exacerbated by its enduring nature, including systematic oppression, economic hardship, violence, human rights violations and national struggle.^2^, ^3^

The groups affected have experienced collective historical traumas. The Jewish people live with stark memories of the Holocaust and centuries of displacement and persecution; and Palestinians have experienced generations of forced displacement, dispossession and oppression living under occupation and precarious socio‐economic conditions.^2^, ^4^ Both groups have been subject to a complex form of transgenerational trauma characterised by a deep sense of injustice and victimisation, promoting feelings of collective resentment, anger and distrust. In that context, collective trauma plays a major role in exacerbating and complicating individual traumatic stress reactions that are triggered by further exposure or reminders of threat to self and others.

Mental distress in these populations can be severe, and trauma of this nature can have deleterious impacts for years to come.^5^ It is instructive to draw on some concrete examples of the immediate stress reactions from our current longitudinal mental health study of 1335 women from refugee background (the WATCH cohort) and its research assistants, more than half of whom are from the Middle East, including from Lebanon and Palestine.^6^, ^7^ All quotes below from study participants have been deidentified and approved by the person who shared it with us.

It is vital for health professionals and support agencies to be aware of the wide‐ranging stress responses and indirect expressions of these reactions that occur among people from migrant and refugee backgrounds. There are various types of reactions that reflect underlying stress in community members. Graphic news stories, social media reports, racial abuse and inadequate public recognition for community level distress can be major sources for mental disturbance.

## Traumatic responses to war

Trauma is described as witnessing or experiencing an extreme stress that overwhelms a person's ability to cope or contradicts one's worldview.^8^ Studies of war‐affected populations show an increase in the incidence and prevalence of mental disorders.^1^ Women are more affected than men, and other groups vulnerable to traumatic stress are children and older people.^1^, ^9^ Prevalence rates in conflict‐affected populations are associated with the degree of trauma, and the degree of access to psychosocial supports.^1^ Harvard Professor of Psychiatry Richard Mollica, in his work with refugees exposed to war, describes trauma as an “invisible wound”, denoting it as a stress reaction following a severe threat to a person's health and life, but often one that is relatively hidden and easily ignored.^10^ War and conflict‐related trauma occurs in its primary form from exposure to or direct involvement in violent conflict, threat to one's life, witness to the death or loss of loved ones and compatriots, fear (including from bombs exploding and the ensuing devastation), rape and sexual assault, kidnapping, torture and arbitrary detention, dislocation from home, and shortage of essentials including food, water and medicine.^5^, ^11^

Secondary traumatic stress, often conflated with the term “vicarious trauma”, results from hearing about or seeing images of first‐hand trauma experienced by another person. Although the literature commonly discusses such stress responses among therapists or first responders assisting trauma‐affected individuals, the risk for trauma symptomology applies to any person connected to the event.^12^, ^13^ The closer one is to identifying with the individual or group, such as a family member or a home village, the higher the risk for adverse psychological impact.^14^ The psychological response to trauma that is either from direct or secondary exposure can include unwanted and intrusive memories of past traumatic events, sleep disturbance, avoidance, and nightmares. These symptoms characterise post‐traumatic stress disorder (PTSD), which is a condition that can be debilitating and follows acute exposure to trauma, in some cases remaining or re‐emerging many years after the traumatic event.^15^ Complex PTSD is particularly relevant to the Israel–Gaza war because it is characterised by prolonged and repeated traumatic incidents that occur over a long period. Complex PTSD has added features including negative impact on self‐esteem and emotional dysfunction.^13^, ^16^ Prolonged grief, depression, anxiety and somatisation can co‐occur in trauma‐affected individuals.^17^ Incidents that are intentional, criminal or unjust are strongly associated with anger and resentment as well as grief and despair.^18^ We note that normal grief and anger responses among those impacted by secondary trauma can be exacerbated by feelings of victimisation, marginalisation and stigmatisation at the community level.

Health professionals may expect to see a higher number of presentations with acute traumatic disturbance, re‐emergence of previously controlled mental illness, and presentations for somatic and unexplained illnesses. On watching news of the Israel–Gaza conflict, a participant from our WATCH study,^6^ from Lebanon, spontaneously remembered Israeli attacks on her village and said: “This made me recall exactly where I was sitting at that time we were bombed, as a child, wondering why this is happening to us, and why can other people have a normal life. It is like I know what they feel.” Other spontaneous descriptions from the study include “that deep feeling of fear when we could hear the explosions, then the bomb hit the side of our shelter, we ran barefoot into the street to escape”, and “remembering my child's face and seeing children of the same age now, I feel frozen with helplessness”. These reactions echo a body of knowledge and evidence describing the psychological response being strongly tied to the re‐emergence of traumatic memory, the embodied and visceral recollection of fear, loss and helplessness. Trauma‐related impairment of coping and functioning was articulated by another participant who said: “It is like I am walking in slow motion; I am not happy as I was, it is burning me inside.”

Secondary traumatic responses can also include externalised expressions of anger as well as intense feelings of separation anxiety from members of the immediate family. A participant from the affected region said: “I send my children off to school, but I think about them all day, and worry about them excessively. This has not happened to me in this way before.”

## How should health professionals respond?

In the tradition of a trauma‐informed care approach, health professionals should be mindful of the various manifestations of trauma, and how they may be impacting people's lives.^19^ Patients may not be aware that their presenting symptoms are related to the current trauma, or they may fear disclosing their concerns about the war in case it is met with an adverse or unsupportive response. Direct questions to adults about how the conflict is affecting them are usually preferred over indirect questions about how they are feeling. Health professionals should explore the psychological and physical effects of traumatic stress on the client and their children, being sensitive to the patient's tolerance of how much they can disclose without losing control of their emotions. It is empowering to reassure clients that they are not alone in feeling this way, and that many others are finding this a stressful and harrowing time. Make a referral for specifically targeted services if required. Advise clients not to focus excessively on social and other media, and to limit exposure to a window of one or two hours daily.^20^ Remind them to ensure media sources are accurate and informed. Encourage people to stay connected with family and friends and use exercise and lifestyle strategies to reduce stress, including meditation and relaxation. Although they are often overlooked, cultural and religious coping strategies are vital in providing comprehensive and strengths‐based community care.^1^, ^21^ Our current experience conducting refugee‐focused research further unveiled the acute risk of secondary trauma among practitioners and researchers listening to trauma stories. Secondary traumatic responses are compounded for practitioners and researchers who have lived in war zones or have personal connections to the war. These “wounded healers” need to be supported and encouraged to care for their mental wellbeing and seek professional interventions if needed.^22^

A broad public health position that extends beyond attending to the trauma responses among individuals is for health and social leaders at the community level to be advocates. Advocates for mental health during this traumatic period should advise against taking a partisan position, and instead promote an understanding of human rights, historical trauma, and current mental distress among those affected by the war. The consequences of not advocating for bipartisan human rights has already been felt in Australia by way of racialised hate speech and racism, including anti‐Semitic sentiment and verbal assaults targeting women wearing hijabs.^23^, ^24^ It is critical for community leaders to demonstrate and advocate for a measured public response to this war, promoting understanding, validation, and the need to maintain order and peace in our country.

We are a multicultural country with large populations of people who will be seriously impacted by the trauma of this war that has been occurring in their homeland, or because the region has political, cultural or religious significance to them. Health professionals need to be aware of the complexity of the traumatic stress reactions that conflict‐affected communities can manifest under these circumstances, and the importance of offering multilevel interventions to address these reactions, drawing on all the resources available to provide support for patients and their families.

## Open access

Open access publishing facilitated by University of New South Wales, as part of the Wiley ‐ University of New South Wales agreement via the Council of Australian University Librarians.

## Competing interests

No relevant disclosures.

## Provenance

Not commissioned; externally peer reviewed.

## References

[1] Murthy RS , Lakshminarayana R . Mental health consequences of war: a brief review of research findings. World Psychiatry 2006; 5: 25‐30. 16757987 PMC1472271

[2] Helbich M , Jabr S . A call for social justice and for a human rights approach with regard to mental health in the occupied Palestinian territories. Health Human Rights J 2022; 24: 305‐318. PMC9790960 36579325

[3] Lavi I , Canetti D , Sharvit K , et al. Protected by ethos in a protracted conflict? A comparative study among Israelis and Palestinians in the West Bank, Gaza, and East Jerusalem. J Conflict Resolution 2012; 58: 68‐92.

[4] Diab M , Veronese G , Abu Jamei Y , et al. Psychosocial concerns in a context of prolonged political oppression: Gaza mental health providers’ perceptions. Transcult Psychiatry 2023; 60: 577‐590. 34986045 10.1177/13634615211062968

[5] Silove D , Ventevogel P , Rees S . The contemporary refugee crisis: an overview of mental health challenges. World Psychiatry 2017; 16: 130‐139. 28498581 10.1002/wps.20438 PMC5428192

[6] Rees S , Mohsin M , Moussa B , et al. Cohort profile: intimate partner violence and mental health among women from refugee background and a comparison group of Australian‐born – the WATCH cohort study. BMJ Open 2022; 12: e051887. 10.1136/bmjopen-2021-051887 PMC9086637 35534066

[7] Rees SJ , Fisher JR , Steel Z , et al. Prevalence and risk factors of major depressive disorder among women at public antenatal clinics from refugee, conflict‐affected, and Australian‐born backgrounds. JAMA Netw Open 2019; 2: e193442. 31050785 10.1001/jamanetworkopen.2019.3442 PMC6503483

[8] Horowitz M. Treatments of psychiatric disorders: a task force report of the American Psychiatric Association. Washington, DC: American Psychiatric Association, 1989.

[9] Thabet AM , Thabet SS , Vostanis P . The relationship between war trauma, PTSD, depression, and anxiety among Palestinian children in the Gaza Strip. Health Sci J 2016; 10: 1‐8.

[10] Mollica RF . Waging a new kind of war. Invisible wounds. Sci Am 2000; 282: 54‐57. 10862423

[11] Silove D , Liddell B , Rees S , et al. Effects of recurrent violence on post‐traumatic stress disorder and severe distress in conflict‐affected Timor‐Leste: a 6‐year longitudinal study. Lancet Glob Health 2014; 2: e293‐e300. 25103168 10.1016/S2214-109X(14)70196-2

[12] Lev‐Wiesel R , Amir M . Secondary traumatic stress, psychological distress, sharing of traumatic reminiscences, and marital quality among spouses of Holocaust child survivors. J Marital Fam Ther 2001; 27: 433‐444. 11594012 10.1111/j.1752-0606.2001.tb00338.x

[13] Lehrner A , Yehuda R . Trauma across generations and paths to adaptation and resilience. Psychol Trauma 2018; 10: 22‐29. 29323523 10.1037/tra0000302

[14] Hensel JM , Ruiz C , Finney C , Dewa CS . Meta‐analysis of risk factors for secondary traumatic stress in therapeutic work with trauma victims. J Trauma Stress 2015; 28: 83‐91. 25864503 10.1002/jts.21998

[15] Yehuda R. Post‐traumatic stress disorder. N Engl J Med 2002; 346: 108‐114. 11784878 10.1056/NEJMra012941

[16] Silove D , Tay AK , Kareth M , Rees S . The relationship of complex post‐traumatic stress disorder and post‐traumatic stress disorder in a culturally distinct, conflict‐affected population: a study among West Papuan refugees displaced to Papua New Guinea. Front Psychiatry 2017; 8: 73. 28620322 10.3389/fpsyt.2017.00073 PMC5449451

[17] Schauer M , Schauer E . Trauma‐focused public mental‐health interventions: a paradigm shift in humanitarian assistance and aid work. In: Martz E , editor. Trauma rehabilitation after war and conflict. Springer, 2010; pp. 389‐428.

[18] Rees S , Silove D . Sakit hati: a state of chronic mental distress related to resentment and anger amongst West Papuan refugees exposed to persecution. Soc Sci Med 2011; 73: 103‐110. 21665342 10.1016/j.socscimed.2011.05.004

[19] Benjamin R , Haliburn J , King S , editors. Humanising mental health care in Australia: a guide to trauma‐informed approaches. Oxfordshire, UK: Routledge, 2019.

[20] Wiederhold BK . A legacy of trauma: how local conflicts can have global implications for mental health. Cyberpsychol Behav Soc Netw 2023; 10.1089/cyber.2023.29296.editorial [Epub ahead of print]. 37751187

[21] Menyhért A. Digital trauma processing in social media groups: transgenerational Holocaust trauma on Facebook. Hungarian Historical Rev 2017; 6: 355‐376.

[22] Mollica R. Healing invisible wounds: paths to hope and recovery in a violent world. Nashville, TN: Vanderbilt University Press, 2008.

[23] Selod S. Citizenship denied: the racialization of Muslim American men and women post‐9/11. Critical Sociol 2014; 41: 77‐95.

[24] Staszewska E. As the Hamas‐Israel war rages, Islamophobia and antisemitism are rising in Australia. SBS News, 28 Oct 2023. https://www.sbs.com.au/news/article/as‐the‐hamas‐israel‐war‐rages‐islamophobia‐and‐antisemitism‐are‐rising‐in‐australia/385jt872y (viewed Nov 2023).

